# Dissociable and Paradoxical Roles of Rat Medial and Lateral Orbitofrontal Cortex in Visual Serial Reversal Learning

**DOI:** 10.1093/cercor/bhz144

**Published:** 2019-07-25

**Authors:** M E Hervig, L Fiddian, L Piilgaard, T Božič, M Blanco-Pozo, C Knudsen, S F Olesen, J Alsiö, T W Robbins

**Affiliations:** 1 Department of Psychology, University of Cambridge, Cambridge, UK; 2 Behavioral and Clinical Neuroscience Institute, University of Cambridge, Cambridge, UK; 3 Department of Neuroscience, University of Copenhagen, Copenhagen N, Denmark; 4 Research Laboratory for Stereology and Neuroscience, Copenhagen University Hospital, Bispebjerg, Copenhagen NV, Denmark

**Keywords:** amygdala, orbitofrontal cortex, prefrontal cortex, reversal learningm, visual discrimination

## Abstract

Much evidence suggests that reversal learning is mediated by cortico-striatal circuitries with the orbitofrontal cortex (OFC) playing a prominent role. The OFC is a functionally heterogeneous region, but potential differential roles of lateral (lOFC) and medial (mOFC) portions in visual reversal learning have yet to be determined. We investigated the effects of pharmacological inactivation of mOFC and lOFC on a deterministic serial visual reversal learning task for rats. For reference, we also targeted other areas previously implicated in reversal learning: prelimbic (PrL) and infralimbic (IL) prefrontal cortex, and basolateral amygdala (BLA). Inactivating mOFC and lOFC produced opposite effects; lOFC impairing, and mOFC improving, performance in the early, perseverative phase specifically. Additionally, mOFC inactivation enhanced negative feedback sensitivity, while lOFC inactivation diminished feedback sensitivity in general. mOFC and lOFC inactivation also affected novel visual discrimination learning differently; lOFC inactivation paradoxically improved learning, and mOFC inactivation had no effect. We also observed dissociable roles of the OFC and the IL/PrL. Whereas the OFC inactivation affected only perseveration, IL/PrL inactivation improved learning overall. BLA inactivation did not affect perseveration, but improved the late phase of reversal learning. These results support opponent roles of the rodent mOFC and lOFC in deterministic visual reversal learning.

## Introduction

The fundamental ability to flexibly change behavior in response to situational changes is disrupted in several psychiatric and developmental disorders including obsessive compulsive disorder (OCD), schizophrenia, and autism (Waltz and Gold 2007; Chamberlain et al. 2008; Leeson et al. 2009; D’Cruz et al. 2013). Reversal learning paradigms are commonly used to assess flexible responding to changing reinforcement contingences in humans (Murphy et al. 2002; Fellows and Farah 2003), monkeys (Butter 1969; Dias et al. 1996; Groman et al. 2013), and rodents (Chudasama and Robbins 2003; McAlonan and Brown 2003). In reversal learning, initially learned reward contingencies are switched and the subject needs to update behavior accordingly. This requires different cognitive processes including the ability to suppress the tendency to persist with the previously rewarded response, learning the new contingencies, and choosing the previously unrewarded (but now rewarded) option. Failure to adapt behavior often manifests as increased perseverative responding (Iversen and Mishkin 1970).

A vast amount of work across species suggests that reversal learning is mediated by cortico-striatal circuitries with the orbitofrontal cortex (OFC) playing a key role (Izquierdo et al. 2017). In humans, reversal learning activates the OFC (O’Doherty et al. 2001; Hampshire and Owen 2006; Ghahremani et al. 2010) and OFC damage impairs discrimination reversal learning though not initial acquisition (Rahman et al. 1999; O’Doherty et al. 2001; Fellows and Farah 2003; Hornak et al. 2004). Whereas there is some evidence against a specific role of the macaque OFC in reversal learning (Rudebeck et al. 2013b), a more posterolateral region has been implicated (Chau et al. 2015). The OFC is critical for reversal learning in marmoset monkeys (Dias et al. 1996; Clarke et al. 2008) and a vast amount of evidence implicates the lateral OFC (lOFC) in rodents (Schoenbaum et al. 1999, 2000, 2003; Bohn et al. 2003; McAlonan & Brown 2003; Kim & Ragozzino 2005; Burke et al. 2009; Takahashi et al. 2009; see review by Izquierdo et al. 2017). However, the OFC is a heterogeneous region (Izquierdo 2017) and functional dissociations have been shown between the rodent lOFC and medial OFC (mOFC) in cocaine-seeking behavior (Fuchs et al. 2004), delay-discounting with spatial reversal (Mar et al. 2011), and probabilistic spatial reversal learning (Dalton et al. 2016). Although lOFC inactivation (Alsiö et al. 2015) and excitotoxic lesioning (Graybeal et al. 2011) impair deterministic visual serial reversal learning in rodents, the effects of mOFC inactivation have not previously been determined in this setting.

Consequently, we compared the effects of inactivating these structures on deterministic visual reversal learning in rats. We employed a touchscreen paradigm as used for humans (Rahman et al. 1999) and included serial reversals as also used in human imaging studies (Cools et al. 2009; Ghahremani et al. 2010) to establish the principle or rule of reversal learning (Rygula et al. 2010), and to achieve within-subject reversal learning performance, suitable for assessing acute manipulations. We hypothesized different, and even opposite, effects of lOFC and mOFC inactivations on reversal learning given apparent functional dissociations between the human lOFC and mOFC in, for example, OCD (see reviews: Menzies et al. 2008; Milad and Rauch 2012; Fettes et al. 2017; Robbins et al. 2019) and rodent optogenetic studies showing stimulation of mOFC (Ahmari et al. 2013) and lOFC (Burguière et al. 2013) to generate and suppress, respectively, compulsive behavior. We also included a test of novel visual discrimination learning to determine the specificity of any effects on serial reversal learning.

The medial prefrontal cortex (mPFC) has also been associated with aspects of reversal learning (Bussey et al. 1997; Chudasama & Robbins 2003; Graybeal et al. 2011; McAllister et al. 2015; Dalton et al. 2016; Latif-hernandez et al. 2016), although other studies have found less evidence for such involvement (Ragozzino et al. 1999; McAlonan and Brown 2003; Bissonette et al. 2008). Since many of these studies did not differentiate between prelimbic (PrL) and infralimbic (IL) areas, and because effects of inactivation of these structures on visual serial reversal learning do not appear to have been investigated previously, we also inactivated the PrL and IL cortex. Similarly, we investigated effects of inactivation of the basolateral amygdala (BLA) in view of its likely interactions with the OFC (Stalnaker et al. 2007a) and mPFC (Heidbreder and Groenewegen 2003; Chang and Ho 2017). These additional investigations also provided neuroanatomical controls for the comparison with the effects of lOFC and mOFC inactivations.

## Methods and Materials

### Animals

**Figure 1:**
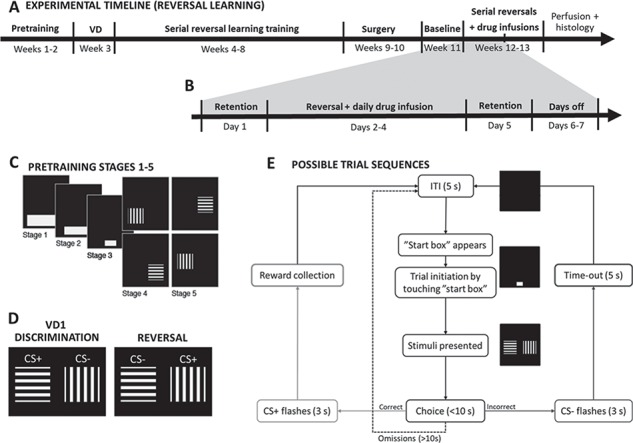
Experimental design—serial visual reversal learning. (*A*) Timeline of the touchscreen serial visual reversal learning experiment (RL) involving behavioral training, surgery and behavioral testing with intracerebral infusions of baclofen/muscimol or vehicle. (*B*) Timeline of one of the two weeks of reversal learning testing with baclofen/muscimol or vehicle infusions. (*C*) Diagram of stimuli presented at different stages of pretraining (from stages 1 to 5). (*D*) Representation of the stimuli presented to the rats during the serial reversal learning training and testing (VD1). (*E*) Flowchart of possible trial sequences in the touchscreen visual discrimination and reversal learning task. CS, conditioned stimulus; ITI, inter-trial interval; VD, visual discrimination.

This research has been regulated under the Animals (Scientific Procedures) Act 1986 Amendment Regulations 2012 (Project license 70/7548) following ethical review by the University of Cambridge Animal Welfare and Ethical Review Body. Male Lister-hooded rats (*N* = 86; Charles River) were allowed to acclimatize to the animal facility for at least 7 days before pretraining commenced. The rats were housed in groups of 4 during the behavioral pretraining period. Following surgical implantation of guide cannulae, the rats were singly housed to protect the implant. Animals were food-restricted with ad libitum access to water, and their body weights were maintained at about 85% of their free-feeding weight. Animals were fed once a day at random times after testing to prevent the animals from anticipating food at certain times. Rats were housed in a temperature- and humidity-controlled environment and maintained under a reverse 12-h light/dark cycle, with lights on at 7 PM. Training and testing occurred during the dark phase. Animals failing to complete any stage of the experiments or with cannula misplacement were excluded from the analysis; see Experimental Design and Statistical Analyses, Figures 1+5, and Supplementary Table S1.

### Drugs

Baclofen hydrochloride (Sigma-Aldrich) and muscimol hydrobromide (Sigma-Aldrich) were dissolved separately in sterile saline and prepared as a baclofen/muscimol mixture with each drug at a final concentration of 1.0 mM as in (Zeeb et al. 2010; Alsiö et al. 2015) for infusions in prefrontal cortex (PFC) subregions. For BLA infusions the baclofen/muscimol mixture was prepared in the same way, but with a 10:1 factor between baclofen and muscimol (as in Yu & Sharp 2015) to a final concentration of 0.1/0.01 mM baclofen/muscimol. Drug doses were optimized for each brain region, and doses on which the rats could complete the task (>200 trials) were chosen. Aliquots were frozen at −80°C in the quantities required for each test day. For intra-cranial microinfusions, baclofen/muscimol was administered at a volume of 0.5 μL/side 10 min prior to testing.

### Behavioral Training (Touchscreen Serial Visual Reversal Learning)

This paradigm was designed as a serial reversal learning task with consistent perseverative behavior across reversals to allow within-subject pharmacological assessment in rats. Task parameters such as stimuli, criteria for perseveration and learning, number of retention sessions between reversals, etc. were previously defined and validated (Alsiö et al. 2015). For experimental timeline and design, see Figure 1.

### Apparatus

For training and testing, we used 16 operant chambers (Med Associates) with dimensions 30 × 39 × 29 cm and a Perspex ceiling, front door and back panel, and metal paneling on the sides of the chamber. The floor of the chamber was covered with a metal grid with a metal tray beneath. The operant chambers were placed in sound- and light-attenuating wooden boxes with fans for the purpose of ventilation and masking external noise. In each box, a central food magazine with light and infrared beam to detect entries was connected to an external pellet dispenser delivering one 45 mg sucrose pellet at a time (TestDiet 5TUL; Sandown Scientific). A house-light (~3 W) was located near the ceiling directly above the magazine. The opposite side of the chamber contained a touch-sensitive screen (dimensions: 29 x 23 cm) presenting 2 stimuli at a time. Task schedules were developed and implemented by Dr A.C. Mar using Visual Basic 2010 and has been published previously (Alsiö et al. 2015).

### Pretraining—Touchscreen Serial Visual Reversal Learning

Shortly after food restriction, the rats underwent 5 pre-training stages (Fig. 1C) involving Pavlovian and instrumental conditioning before moving on to visual discrimination learning followed by serial reversals until stable baseline was reached. Rats responded at a single white box displayed on the touch-sensitive screen (“start box”) taking up nearly its whole bottom centre, for sucrose reward pellets during 60-min daily sessions until the rat reached the criterion of receiving maximum 100 pellets in 1 session. When criterion was reached the rat moved on to the next pre-training stage, where the size of the white box was reduced to an intermediate size (pre-training stage 2) and the final size of 3 × 4 cm (pre-training stage 3). At pre-training stages 4 and 5, 2 stimuli were introduced (horizontal and vertical bars). Touching the white start box was no longer reinforced, but instead led to the presentation of one of these stimuli to the left or right in a pseudo-random order—located near the bottom of the screen. Responding to this stimulus was reinforced with a sugar pellet, whereas responding to the blank side was signaled as incorrect by the illumination of the house-light for a 5 s time-out period. After the rat had reached ≥80% correct touches on one stimulus, it moved to sessions with the alternative stimulus. When criterion was reached also on this stimulus, the rats moved on to next stage (stage 5), where the position of the stimuli was raised approximately 5 cm on the screen, to the final position, in order to avoid accidental touches. The single stimulus presented was horizontal or vertical bars on alternate days as in stage 4. After ≥80% correct touches were reached on both stimuli, visual discrimination training ensued.

### Visual Discrimination Training

Visual discrimination training was similar to stage 5, but the rats were presented with both stimuli simultaneously. For trial initiation, the rats responded to the white start box at the bottom centre of the screen followed by simultaneous presentation of the visual discrimination stimuli pair (VD1; Fig. 1D). One conditioned stimulus (CS) was reinforced (CS+) with a sugar pellet, while touches on the non-reinforced stimulus (CS−) would initiate a house-light-signaled 5 s time-out period. Failure to make a choice of either stimulus within the 10 s limited hold caused both stimuli to be removed from the screen and the trial was recorded as an omission. A 5 s inter-trial interval followed each trial. The position of the 2 stimuli were presented on the screen in a pseudo-random order (max. 3 consecutive trials to the same side) to prevent the rats from developing a side bias. The daily session ended after 60 min, 150 rewards or 250 trials, whichever occurred first. When the rats reached the discrimination criterion of 24 correct out of a running window of 30 trials, the rat moved on to serial reversal learning training.

### Serial Visual Reversal Learning

Once discrimination was acquired, rats were given a retention session the following day using the same reward contingencies to confirm that the rats had acquired the discrimination. Following the retention session, the contingencies reversed and the rats were required to respond to the previous CS− (now CS+) until they reached the reversal learning criterion (24/30 correct responses). A retention session was always performed on the day before each reversal and on the day after criterion was met (Fig. 1B). Thus, one reversal followed the following schedule: retention day (CS+, CS−), reversal day 1 (CS−, CS+), reversal day 2 (CS−, CS+), reversal day 3 (CS−, CS+),…etc. (until learning criterion was reached), retention day (CS−, CS+) (see also Fig. 1B). Additional reversals [back to (CS+, CS−) a.o.] were performed until the rats were able to reach the criterion within three daily sessions with more than 200 trials completed on the first reversal day. When this criterion was met, the rat underwent surgery (see Fig. 1A).

### Serial Novel Visual Discrimination Learning

To investigate whether drug effects in the mOFC and lOFC were selective for reversal learning and not discrimination learning acquisition per se, 2 other groups of rats were tested with 2 sets of novel visual discriminanda (VD2 and VD3; Fig. 5C) following serial reversal training (with VD1 stimuli as described above) and cannulation (for timeline, see Fig. 5A+B), where 1 stimulus was rewarded and the other was not (counter-balanced). Once they reached criterion (24/30), they received 2 retention sessions followed by presentation of the other novel stimuli pair.

### Stereotaxic Surgery

Rats were anesthetized (isoflurane induced at 5% and maintained at 2%) and secured in a stereotaxic frame (KOPF) with atraumatic ear bars. The tooth bar was set to −3.3 mm and adjusted for flat skull position. Bilateral guide cannulae (22-GA; PlasticsOne) were implanted in the PrL or IL [anteroposterior (AP) +2.7, mediolateral (ML) ±0.75, dorsoventral (DV) −1.0), lOFC (AP +3.5, ML ±2.5, DV −1.7), mOFC (AP +4.0, ML ±0.6, DV −1.4) or BLA (AP −2.6, ML ±4.5, DV −2.5) and secured with 4 screws and dental cement. Obdurators ending flush with the guide cannulae were inserted and protected with a dust cap. Surgical coordinates were obtained using a stereotaxic atlas (Paxinos and Watson 2004) and further adjusted according to pilot surgeries. AP and ML coordinates were referenced to Bregma and DV was referenced to dura.

### Intracerebral Microinfusions

After recovery from surgery (≥7 days), behavioral training resumed to re-baseline the rats to ensure stable serial reversal learning performance before microinfusions could begin. The rats received a retention session followed by a reversal the next day without drug infusion. When the criterion was reached, the rats received another retention session. During this baseline reversal, rats were habituated to the infusion procedure and received sham infusions. Following the baseline reversal, rats received intracerebral infusions of the baclofen/muscimol mixture across reversals according to a within-subject, cross-over/Latin-square design. Injectors from PlasticsOne (28-GA) were extended 2 mm (lOFC and mOFC), 2.5 mm (PrL), 3.5 mm (IL), or 6 mm (BLA) below the guide for regional infusions. Drug infusions were performed in a volume of 0.5 μL over 2 min. The injector was left in place for 1 min before and after infusion. During the infusion procedure, the rats were gently restrained or allowed to freely move on the experimenter’s lap. Microinfusions were given each day of the reversal, that is, from the session when contingencies first shifted to the day criterion was reached (Fig. 1A+B). Rats that reached criterion on the third day thus received 3 infusions on three consecutive days during that reversal. Retention sessions (no infusions) were included the day after criterion was met and again before the next reversal started. On the retention session just prior to the reversal, rats received saline infusion to ensure habituation to the infusion procedure. Rats typically had 2 days without testing between these retention sessions (i.e., a full reversal with retention sessions and break took 7 days, during which the rats typically received 3 infusions). For the visual novel discrimination experiment (Fig. 5), the microinfusion and testing procedure was as described above, although the rats would normally reach criterion on the first (and at least on the second) testing day, that is, these rats received 1–2 infusions during one discrimination testing (Fig. 5B).

### Histology

At the end of the experiments, animals were given a lethal dose of sodium pentobarbitone and perfused transcardially with 0.01 M PBS followed by 4% paraformaldehyde. The brains were removed, post-fixed in 4% paraformaldehyde for 24 h and preserved in 30% sucrose in 0.01 M PBS for 2 days until sectioning. For sectioning, the brains were frozen and embedded in optimal cutting temperature compound (VWR Chemicals, #361603E). They were cut into 60-μm coronal sections using a cryostat (Leica, CM3050 S) and systematically sampled in 6 series. The sections were stored in cryoprotectant at −20°C until Cresyl Violet staining to verify regional injector-tip placements.

### Experimental Design and Statistical Analyses

**Figure 2:**
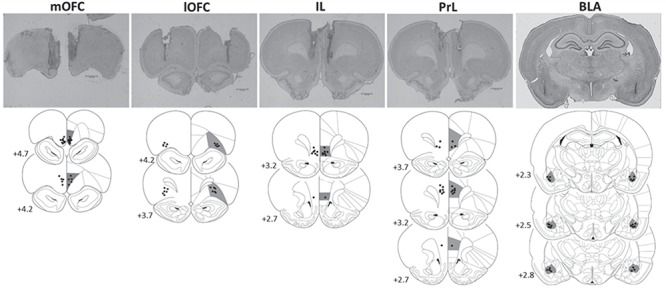
Schematic representation of brain sections showing the infusion sites in the mOFC (*N* = 14), lOFC (*N* = 11), IL (*N* = 8), PrL (*N* = 11), and BLA (*N* = 13) included in the reversal learning analyses. Infusion sites were characterized from brain sections prepared with Cresyl violet. Coordinates are given as millimeter distance from bregma. (Diagrams modified from Paxinos and Watson 2004).

Only animals with intact cannulae during the course of the experiments and with correct regional placement of injector tips (Fig. 2+5D) were included in the analyses (Supplementary Table S1).

All experiments employed a within-subject complete crossover/Latin-square design with separate cohorts for each region. Data from each reversal (or novel discrimination) were collapsed over days. Trial outcomes were next coded as perseverative, random or learning depending on performance over bins of 30 trials in a rolling window (as illustrated in Supplementary Figure S1) and based on binomial distribution probabilities as originally described and employed by Jones and Mishkin (1972). Thus, any error performed within a 30-trial bin in which the rat displayed a significant bias toward the previously correct stimulus (<11 correct) was coded as perseverative, whereas any 30-trial bin in which the rat displayed a significant bias toward the currently correct stimulus (>19 correct) was coded as new learning. When the rat chose either stimulus with approximately equal probability (i.e., 11–19 correct per 30 trials) it was coded as intermediary/random phase. Bins were coded as perseverative, random or learning wherever they occurred during the session, meaning that rats technically could shift multiple times between perseverative and random, and random and learning phases. Post-criterion data (>24 correct) were excluded from analysis.

Behavioral data were subjected to analysis of variance (ANOVA) using a general linear model with significance at α = 0.05. Data were initially tested for normality with the Shapiro–Wilk test and outliers by inspection of studentized residuals. An outlier would only be excluded from the analyses if the subject was consistently an outlier across all drug doses, and no animals were excluded. Homogeneity of variance was verified using Levene’s test. For repeated-measures analyses, Mauchly’s test of sphericity was applied to assure the sphericity assumption was not violated. Data that did not pass the Shapiro–Wilk test was appropriately transformed to obtain normal distribution before analysis.

The dependent variables were errors, reward collection and response latencies, omissions as well as win–stay and lose–shift probabilities. Errors were square-root transformed and analyzed to learning criterion and in each phase across regions. Lose–shift and win–stay probabilities were arcsine transformed an analyzed to criterion. Non-parametric test was applied to analyze omissions to criterion (Wilcoxon) (note that omissions only occurred if the animals actively initiated a trial by touching the “start box”). Latencies to respond at the stimuli (after initiating a trial) and to collect earned reward pellets were analyzed to criterion.

To investigate whether treatment had an impact on the overall learning strategy we additionally analyzed the win–stay and lose–shift behavior as a proxy for learning from positive and negative feedback, respectively. We calculated the win–stay strategy as the probability of making a correct choice after a correct trial (P [stay|win]) and the lose–shift strategy as the probability of making a correct choice after an incorrect trial P [shift|loss] (Clarke et al. 2008; Riceberg and Shapiro 2012). Thus, P [shift|win] + P [stay|win] = 1 and P [shift|loss] + P [stay|loss] = 1.

The “criterion of learning” and “behavioral phase” data analyses across regions were performed with two-way mixed ANOVAs in a within-subject (treatment) × between-subject (region) design for regional inactivation. Data were analyzed within each region using planned pairwise comparisons with Student’s *t*-tests.

All statistical analyses were performed using the SPSS statistical package (IBM SPSS Statistics, Version 25.0.0.1) and graphs were generated using GraphPad Prism 7. Data are presented as mean ± standard error of mean (SEM). *P* < 0.05 will be described as significant, while *P* > 0.1 will be reported as non-effects. Effect sizes are indicated with partial eta-squared (*ηp^2^*) (Cohen 1988).

## Results

### Histological Assessment of Regional Infusion Sites

For cohort details for the reversal learning experiment, see Supplementary Table S1. Of the 71 animals entering the reversal learning experiment, 57 rats were included in the analysis based on histological assessment of regional infusion sites; comprising of 14 (mOFC), 12 (lOFC), 8 (IL), 11 (PrL), and 13 (BLA) rats with correct regional injector placements (Fig. 2). Of the 15 animals entering the novel discrimination experiment, all animals were included: 9 (mOFC) and 6 (lOFC) rats (Fig. 5).

### Effects of mOFC, lOFC, IL, PrL, and BLA Inactivation on Reversal Learning

**Figure 3:**
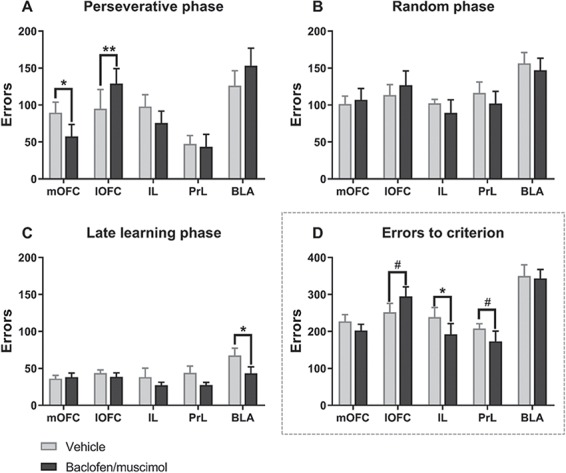
Effects of site-specific pharmacological inactivation on performance in deterministic touchscreen serial visual reversal task. (*A*–*C*) The effect of pharmacological inactivation on errors within each reversal learning phase: perseveration (*A*), random (*B*), and late learning (*C*). OFC inactivation affected only perseverative errors (*A*), with mOFC and lOFC exhibiting dissociable roles; inactivating the lOFC impaired, while mOFC inactivation improved, serial reversal learning performance as reflected by an increase and decrease in number of perseverative errors, respectively. (*D*) The effect of pharmacological inactivation on total errors to criterion of learning. Dissociable roles of the OFC and mPFC (IL and PrL) in deterministic serial visual reversal learning, as OFC inactivation affected only perseveration and mPFC inactivation affected learning overall. Results are represented as mean ± SEM; **P < 0.01; *P < 0.05; #*P* < 0.1. Veh, vehicle; BM, baclofen/muscimol.

Intra-OFC baclofen/muscimol produced contrasting effects on errors, with lOFC inactivation significantly increasing perseverative responses and mOFC inactivation significantly reducing them (Fig. 3A).

For perseverative errors, ANOVA showed a significant inactivation × region interaction (*F*_4, 52_ = 4.11, *P* = 0.006, *ηp^2^* = 0.24) and main effect of region (*F*_4, 52_ = 5.22, *P* = 0.001, *ηp^2^* = 0.29), while there was no main effect of inactivation (*F*_1, 52_ = 0.464, *P* = 0.499, *ηp^2^* = 0.009) (Fig. 3A). Planned pairwise comparisons within each region showed that lOFC inactivation significantly increased the number of errors (*t*_10_ = −3.15, *P* = 0.010, *ηp^2^* = 0.50), while the mOFC significantly decreased number of errors in the perseveration phase (*t*_13_ = 2.52, *P* = 0.026, *ηp^2^* = 0.33). There were no significant effects of inactivating the BLA (*t*_12_ = −0.927, *P* = 0.372, *ηp^2^* = 0.067), IL (*t*_7_ = 1.226, *P* = 0.260, *ηp^2^* = 0.18), or PrL (*t*_10_ = 0.803, *P* = 0.440, *ηp^2^* = 0.061) on perseverative errors.

For the random phase, ANOVA showed a main effect of region (*F*_4, 52_ = 3.188, *P* = 0.020, *ηp^2^* = 0.197), but no inactivation × region interaction (*F*_4, 52_ = 0.316, *P* = 0.866, *ηp^2^* = 0.024) and no main effect of inactivation (*F*_4, 52_ = 0.817, *P* = 0.370, *ηp^2^* = 0.015) (Fig. 3B).

For the late learning phase, ANOVA showed a significant main effect of treatment (*F*_1, 52_ = 6.00, *P* = 0.018, *ηp^2^* = 0.10) and region (*F*_4, 52_ = 2.74, *P* = 0.038, *ηp^2^* = 0.17), but no inactivation × region interaction (*F*_4, 52_ = 1.177, *P* = 0.332, *ηp^2^* = 0.083) (Fig. 3C). Planned pairwise comparisons within each region revealed that inactivating the BLA significantly decreased number of errors in the late learning phase (*t*_12_ = 2.85, *P* = 0.015, *ηp^2^* = 0.40), while there were no effect of inactivating the lOFC (*t*_10_ = 1.02, *P* = 0.33, *ηp^2^* = 0.094), mOFC (*t*_13_ = −0.190, *P* = 0.85, *ηp^2^* = 0.003), PrL (*t*_10_ = 1.43, *P* = 0.183, *ηp^2^* = 0.17), and IL (*t*_7_ = 0.55, *P* = 0.600, *ηp^2^* = 0.041).

For errors to criterion, there was a significant main effect of region (*F*_4, 52_ = 9.87, *P* < 0.001, *ηp^2^* = 0.43) and a trend toward an inactivation × region interaction (*F*_4, 52_ = 2.11, *P* = 0.092, *ηp^2^* = 0.14), and no main effect of inactivation (*F*_1, 52_ = 2.53, *P* = 0.12, *ηp^2^* = 0.046). While inactivating mPFC regions did not affect specific reversal learning phases (Fig. 3A–C), it did reduce errors to criterion (Fig. 3D). Planned pairwise comparisons within each region revealed a decrease in errors to criterion after inactivating the IL (*t*_7_ = 2.36, *P* = 0.050, *ηp^2^* = 0.44), a trend toward decreased errors in the PrL (*t*_10_ = 1.88, *P* = 0.090, *ηp^2^* = 0.26), a trend toward increased errors in the lOFC (*t*_10_ = −2.182, *P* = 0.054, *ηp^2^* = 0.32), and no effects in the mOFC (*t*_13_ = 1.37, *P* = 0.20, *ηp^2^* = 0.13) or BLA (*t*_12_ = 0.095, *P* = 0.93, *ηp^2^* = 0.001).

In sum, pharmacological inactivation of the lOFC and mOFC selectively increased and reduced, respectively, perseveration, without affecting later learning phases. By contrast, the IL and PrL did not affect perseveration, but improved learning overall.

Omissions to criterion were significantly increased by inactivating the IL, but not other regions (Supplementary Table S2).

### Sensitivity to Negative and Positive Feedback

**Figure 4:**
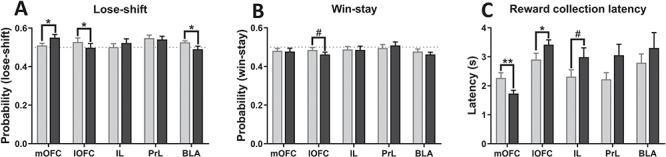
Effects of site-specific pharmacological inactivation on feedback sensitivity and reward collection latency in the deterministic touchscreen serial visual reversal task. Effects of site-specific pharmacological inactivation on the probability to make a correct response after a loss (*A*) and after a win (*B*) as well as on latencies to collect earned food reward (*C*). mOFC inactivation enhanced the sensitivity to negative feedback (trend toward increased lose-shift) and decreased latencies to collect earned food rewards. In contrast, lOFC inactivation produced a diminished sensitivity to both positive and negative feedbacks as well as slower magazine latencies. Results are represented as mean ± SEM; **P < 0.01; *P < 0.05; #*P* < 0.1.

We further investigated whether regional inactivation affected positive or negative feedback sensitivity by evaluating win-stay and lose–shift probabilities. For the lose–shift probability (Fig. 4A), ANOVA revealed a significant inactivation × region interaction (*F*_4, 52_ = 3.30, *P* = 0.018, *ηp^2^* = 0.20) with no main effects of inactivation (*F*_1, 52_ = 0.034, *P* = 0.854, *ηp^2^* = 0.001) or region (*F*_1, 52_ = 1.04, *P* = 0.30, *ηp^2^* = 0.088). Planned pairwise comparisons for each region revealed that the lose–shift probability was significantly increased by mOFC inactivation (*t*_13_ = −2.25, *P* = 0.042, *ηp^2^* = 0.28) and significantly decreased by lOFC (*t*_10_ = 2.24, *P* = 0.049, *ηp^2^* = 0.33) and BLA (*t*_12_ = 2.17, *P* = 0.050, *ηp^2^* = 0.28) inactivation, and was not affected by IL (*t*_7_ = −0.691, *P* = 0.51, *ηp^2^* = 0.064) or PrL (*t*_10_ = 0.407, *P* = 0.69, *ηp^2^* = 0.016) inactivation. For the win–stay probability (Fig. 4B), we found no inactivation × region interaction (*F*_4, 52_ = 0.468, *P* = 0.76, *ηp^2^* = 0.035). However, planned pairwise comparisons within each region revealed that inactivating the lOFC resulted in a trend toward decreased win–stay ratio (*t*_10_ = 1.93, *P* = 0.083, *ηp^2^* = 0.27). Thus, overall we found opposite effects of lOFC and mOFC inactivation on the lose–shift probability, whereas there were no effects after BLA, IL, or PrL inactivation.

### Magazine (Food Reward Collection) and Response Latencies

For reward collection latency (s), there was a significant inactivation × region interaction (*F*_4, 52_ = 2.87, *P* = 0.032, *ηp^2^* = 0.18) with a main effect of inactivation (*F*_1, 52_ = 6.63, *P* = 0.013, *ηp^2^* = 0.11) and region (*F*_4, 52_ = 3.99, *P* = 0.007, *ηp^2^* = 0.24). Planned paired comparisons for each region showed significantly faster reward collection after mOFC inactivation (*t*_13_ = 4.04, *P* = 0.0014, *ηp^2^* = 0.56), and significantly slower reward collection after lOFC inactivation (*t*_10_ = −2.38, *P* = 0.039, *ηp^2^* = 0.36). Inactivating the IL produced a trend toward increase collection latency (*t*_7_ = −2.03, *P* = 0.082, *ηp^2^* = 0.37), while collection latency was not affected by inactivating the PrL (*t*_10_ = −1.72, *P* = 0.12, *ηp^2^* = 0.23) or BLA (*t*_12_ = −1.20, *P* = 0.25, *ηp^2^* = 0.11). We found no effects of regional inactivation on response latencies: no inactivation × region interaction (*F*_4, 52_ = 1.121, *P* = 0.357, *ηp^2^* = 0.079), no main effect of inactivation (*F*_1, 52_ = 0.581, *P* = 0.449, *ηp^2^* = 0.011), and region (*F*_4, 52_ = 0.572, *P* = 0.684, *ηp^2^* = 0.042) (Supplementary Table S2). To explore whether reversal learning effects were correlated with presumable motivational effects, we analyzed the correlation between errors and reward collection latencies. There was a significant positive correlation between number of errors to criterion and reward collection latencies after mOFC inactivation, but no correlations were found with vehicle treatment or inactivation of any other region (Supplementary Table S3).

### Effect of mOFC and lOFC inactivation on novel visual discrimination

**Figure 5:**
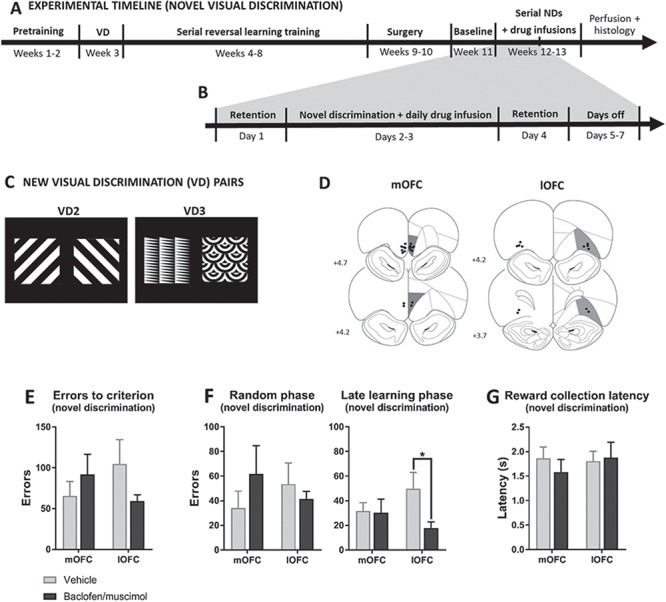
Experimental design and effects of pharmacological inactivation of the mOFC and lOFC on performance in touchscreen serial novel visual discrimination task. (*A*) Timeline of the touchscreen serial novel discrimination experiment involving behavioral training, surgery, and behavioral testing with intracerebral infusions of baclofen/muscimol or vehicle. (*B*) Timeline of one of the two weeks of novel discrimination testing with baclofen/muscimol or vehicle infusions. (*C*) The novel visual discrimination stimuli pairs (VD2 and VD3) that were introduced in the novel discrimination test. (*D*) Baclofen/muscimol infusion sites in the mOFC (*N* = 9) and lOFC (*N* = 6) included in the novel discrimination analyses. Effect of pharmacological inactivation on errors to criterion (*E*) and errors within discrimination phases (*F*). lOFC inactivation decreased learning errors. No effects on reward collection latencies (*G*). Results are represented as mean ± SEM; ^*^P < 0.05.

To investigate the selectivity of reversal learning effects of OFC inactivations, we examined the effects of inactivating the OFC on novel visual discrimination learning (Fig. 5). For number of errors to criterion, we found a trending inactivation × region interaction (*F*_1, 13_ = 3.51, *P* = 0.084, *ηp^2^* = 0.21) with no main effects of inactivation (*F*_1, 13_ = 0.25, *P* = 0.626, *ηp^2^* = 0.019) or region (*F*_1, 13_ = 0.016, *P* = 0.902, *ηp^2^* = 0.001). Planned pairwise comparisons within each region showed no effects. For the effect of inactivation on errors in specific phases of novel discrimination learning (i.e., random and late learning phases), separate two-way repeated-measures ANOVAs within each phase across OFC regions were performed. ANOVA showed no effects in the random phase, but in the late learning phase there was a trending main effect of treatment (*F*_1, 13_ = 3.51, *P* = 0.084, *ηp^2^* = 0.21), but no inactivation × region interaction (*F*_1, 13_ = 2.39, *P* = 0.15, *ηp^2^* = 0.16) or main effect of region (*F*_1, 13_ = 0.129, *P* = 0.725, *ηp^2^* = 0.01). Planned pairwise comparisons showed that lOFC inactivation significantly decreased errors in the late learning phase (*t*_5_ = 3.01, *P* = 0.030, *ηp^2^* = 0.65), while there were no effects of mOFC inactivation (*t*_8_ = 0.228, *P* = 0.825, *ηp^2^* = 0.006) (Fig. 5F). We observed no effects on latencies to collect reward (Fig. 5G), latencies to respond or feedback sensitivity.

### Summary

Results are summarized in Table 1.

**Table 1:** Summary of results

**Region**	**Task**	**To criterion of learning**	**Perseveration** ^**a**^	**Learning** ^**a**^	**Summary**
mOFC	RL	**↑** p [lose-shift]^*^ ↓ Reward collection latency^**^ Positive correlation between errors and collection latency^*^	**↓** Errors^*^	No effect	Improved reversal learning (i.e., decreased perseveration) with increased negative feedback sensitivity and faster reward collection
lOFC	RL	**↑** Reward collection latency^*^ ↓ p [lose-shift]^*^ ↓ p [win-stay]^**#**^ **↑** Errors^**#**^	**↑** Errors^**^	No effect	Impaired reversal learning (i.e., increased perseveration) with diminished feedback sensitivity and slower food collection
IL	RL	**↓** Errors^*^ ↑ Reward collection latency^**#**^	**↑** Omissions^*^	No effect	Improved reversal learning overall
PrL	RL	**↓** Errors^**#**^	No effect	No effect	Trend toward improved reversal learning overall
BLA	RL	**↓** p [lose-shift]^*^	No effect	**↓** Errors^*^	Improved late reversal learning, but decreased negative feedback sensitivity
mOFC	NVD	No effect	N/A	No effect	No effect on NVD learning
lOFC	NVD	No effect	N/A	**↓** Errors^*^	Improved late NVD learning

a ^Note:^ Only the perseveration and late learning phases are included, as there were no effects in the random phase.

N/A, not applicable; NVD, novel visual discrimination; RL, reversal learning. ^**^P < 0.01; ^*^P < 0.05; ^#^*P* < 0.1.

## Discussion

We observed dissociable effects of inactivating OFC and mPFC subregions on deterministic serial visual reversal learning, with OFC inactivation affecting only the perseveration phase and mPFC inactivation improving learning overall. BLA inactivation improved reversal learning significantly in the late stage. Importantly, we found that whereas lOFC inactivation impaired serial visual reversal learning performance by increasing perseverative errors, mOFC inactivation improved it by reducing perseveration. The improved performance after mOFC inactivation was associated with an enhanced sensitivity to negative feedback as reflected by an increased lose–shift trend, and also faster latencies to collect earned food rewards. Conversely, lOFC inactivation diminished sensitivity to negative (and to some extent positive) feedback and produced slower magazine latencies. In contrast to the impairment observed on serial reversal learning following lOFC inactivation, baclofen/muscimol into this area facilitated the learning of visual discrimination with new stimuli after previous serial reversal training training, showing that the reversal learning impairment was not due to general learning deficits. These results add to previous findings showing dissociable roles of the rodent mOFC and lOFC across other tasks such as probabilistic reversal learning (Dalton et al. 2016), delay-discounting (Mar et al. 2011), and instrumental action (Gourley et al. 2010). Although there may be problems in relating rodent OFC regions with those in primates, there is some evidence for homologies (Ongür & Price 2000; Balleine & O’Doherty 2010; Heilbronner et al. 2016), and our findings of dissociable functions of lOFC versus mOFC in the rat are in agreement with studies in humans (Elliott et al. 2000; O’Doherty et al. 2001; Cheng et al. 2016; Noonan et al. 2017) and other primates (Noonan et al. 2010; Walton et al. 2011).

### Effects of Inactivating lOFC on Serial Visual Reversal Learning

The observed impairment in reversal learning following lOFC inactivation is consistent with previous studies involving lOFC inactivation in rats (Kim and Ragozzino 2005; Ragozzino 2007; Alsiö et al. 2015; Dalton et al. 2016) and OFC lesions in monkeys (Dias et al. 1996; Clarke et al. 2008) and rodents (Chudasama and Robbins 2003; McAlonan and Brown 2003; Boulougouris et al. 2007; Bissonette et al. 2008; Riceberg and Shapiro 2012) as well as humans with OFC damage (Rahman et al. 1999; O’Doherty et al. 2001; Fellows and Farah 2003; Berlin et al. 2004; Hornak et al. 2004). Along with the reversal learning impairment, lOFC inactivation reduced sensitivity to both positive and negative feedback, suggesting a deficit in retrieving and incorporating recent information to guide performance, thus resulting in perseveration. This is consistent with human fMRI studies showing that the OFC of healthy subjects represents positive and negative outcome expectancies with the lateral region being more active following a negative outcome (O’Doherty et al. 2001).

In general, previous lOFC lesioning/inactivation studies have shown impairments in reversal learning, but reported no effect on acquisition of new contingencies. We also used a separate novel visual discrimination task following serial reversal training to test learning capacity for new contingencies after lOFC inactivation, and found no effect on acquisition overall, although lOFC inactivation did actually facilitate performance specifically in the late learning phase of this task. This suggests that the reversal learning impairment following lOFC inactivation was likely not due to a general learning deficit, as the rats could acquire novel stimulus–action–outcome contingencies.

The present pattern of findings for lOFC inactivation is difficult to accommodate by existing theories (Dolan and Dayan 2013; Wilson et al. 2014; Domenech and Koechlin 2015; Sharpe et al. 2019). For example, our data might suggest that, following lOFC inactivation, rats place more emphasis on the previous history of reinforcement rather than on recent feedback in making their choices in a reversal task, supporting a role for the lOFC in inhibiting prepotent responses (Man et al. 2009). Consistent with this is the fact that when previous reinforcement history associated with the previous discriminanda were removed there were no deficits in novel discrimination learning. However, this does not immediately explain why there was a significant improvement in new learning, which we will attempt to explain below.

Recent studies have shown that populations of lOFC neurons exhibit task-dependent and reversal-learning phase-dependent firing patterns (Gremel and Costa 2013; Marquardt et al. 2017), which would support different effects of lOFC inactivation in tasks requiring different levels of goal-directed action (Gremel and Costa 2013). The lOFC has been suggested to regulate the balance between goal-directed and habitual learning via interactions with the dorsal striatum in humans (see review by Balleine & O’Doherty 2010; Morris et al. 2016; Gillan et al. 2015), monkeys (Groman et al. 2013), and mice (Gremel and Costa 2013). In particular, the dorsolateral striatum (DLS) is thought to mediate habitual responding (Yin et al. 2004; Yin et al. 2006), with the lOFC controlling striatal activity to inhibit habit learning and promote goal-directed action (Burguière et al. 2013; Gremel and Costa 2013), possibly through lOFC control of local striatal circuits (Burguière et al. 2013) via lOFC NMDA receptor mediated mechanisms (Marquardt et al. 2019). DLS activity is also critical for visual discrimination learning, especially in the later phase, as shown by the lesioning (Brigman et al. 2013) and optogenetic silencing of DLS neurons (Bergstrom et al. 2018). Assuming that our novel visual discrimination task is similarly dependent on the DLS, then the improvement following lOFC inactivation might reflect the removal of an lOFC regulatory influence on the DLS. Therefore, it is conceivable that the lOFC, through its control over DLS, mediates in part a balance between goal-directed and habitual learning, promoting the former while inhibiting the latter, thereby accounting for the significantly improved visual discrimination learning, yet impaired serial reversal performance following lOFC inactivation.

More specifically, the role of the lOFC in goal-directed behavior may extend to strategies of exploitation and exploration of the reinforcement contingencies that have evolved for appropriately adapting behavior in changing situations to enable optimal foraging (Cohen et al. 2007; Domenech and Koechlin 2015). Therefore, it could be postulated that the lOFC is especially implicated in exploration-type strategies that are necessary for discovering the novel contingencies that operate in reversal learning, whereas exploitation strategies hypothetically may be more important for new visual discrimination learning.

lOFC inactivation also had an apparent independent effect to retard the collection of earned food rewards in reversal learning (though not in novel discrimination learning). It is possible this reflects basic impairments in Pavlovian approach responses elicited by CS outcome associations given effects of lOFC lesions on Pavlovian conditioning (Chudasama and Robbins 2003; Ostlund and Balleine 2007). However, this is presumably not a general motivational impairment, but may reflect an impaired anticipation of the rewarding feedback, perhaps arising from increased uncertainty of the outcome of the touchscreen response during reversal.

### Effects of Inactivating mOFC on Serial Visual Reversal Learning

Inactivating mOFC facilitated visual reversal learning performance preferentially in the early, perseverative phase, markedly contrasting with the inactivation of lOFC. This improvement was accompanied by increased sensitivity to negative feedback, and by faster reward collection (possibly reflecting the overall better choice performance after mOFC inactivation, or otherwise increased choice confidence in these rats, maybe due to increased motivational influence), symmetrically with respect to the opposite effects of lOFC inactivation and presumably reflecting contrasting effects on the same hypothesized processes. In contrast with lOFC inactivation, therefore, it could be hypothesized that mOFC inactivation blunts habitual control and thereby improves serial reversal learning, which could also be accounted for by a postulated role of the human mOFC in exploitation processes (Domenech and Koechlin 2015). This theory proposes that the ventral mPFC (including the mOFC) is active during decisions to detect consistencies between expected and actual outcomes according to prepotent stimulus–response mappings (or “task-sets”). Inconsistencies lead to decreased mOFC activation, dorsal mPFC regions (i.e., rodent IL/PrL) then control the switches from exploiting this task set to exploring others. Thus, inactivating the mOFC in our paradigm may switch behavior toward being more exploratory and thus less habitual.

Only a few studies have previously examined the role of the mOFC in reversal learning. These reported either no effect (Dalton et al. 2016) or mOFC-lesion induced perseveration at the previously rewarding location (Gourley et al. 2010) in deterministic spatial reversal. Dalton et al. (2016) further showed impairment in probabilistic serial spatial reversal. The obvious difference is the use, in the present study, of the visual touchscreen reversal paradigm (as opposed to spatial), which requires more training for the rat and may implicate Pavlovian approach responses to a greater extent. Clearly, manipulations of the mOFC generally produce a range of impairments, which, however, can produce incidental benefits in certain situations (Mar et al. 2011; Münster and Hauber 2017). Thus, inactivation/lesioning may have impairing or apparently paradoxical, beneficial, effects depending on the situation (c.f., Young & Shapiro 2009; Riceberg & Shapiro 2017).

### Opponent Functions of lOFC and mOFC

The apparent contrasting functions in serial reversal learning of lOFC and mOFC suggest a competitive balance between these 2 subregions, consistent with anatomical evidence that they are important nodes in independent neural systems (Price 2007; Hoover and Vertes 2011), which may extend into the striatal domains. Our results on serial visual reversal learning could support a notion that mOFC plays a role in retrieval of previous action–outcome associations (Bradfield et al. 2015), consistent with a role for the mOFC in associative memory (reviewed in, e.g., Pergola & Suchan 2013). When inactivating the mOFC, past history will not interfere with representation of current states and thus behavior is more readily updated. Conversely, the lOFC has been suggested to represent the “current state” (Wilson et al. 2014; Sharpe et al. 2019)—consistent with a role in working memory (e.g., Wallis 2007). Inactivating the lOFC may remove a control over history interfering with current states and the animal will not be able to properly update behavior, thus resulting in perseveration. A functional interaction between the mOFC and lOFC could mediate the balance between these two “systems”, that is, a “memory system” represented by the mOFC and a “current state system” represented by the lOFC. However, it is again difficult to understand how this could explain why lOFC inactivation enhances novel visual discrimination learning, as this should require an update of the “current state” by the lOFC.

Alternatively, the functional balance between mOFC and lOFC could be understood in terms of “explore versus exploit” strategies described above (Cohen et al. 2007; Domenech and Koechlin 2015). Thus, inactivating the mOFC may facilitate exploration mediated by the lOFC that is now unrestricted by the mOFC; diminishing exploitation of the previous stimulus–reward association promotes switching to the new association, thus improving performance. Conversely, lOFC inactivation reduces exploration, which increases the likelihood of committing incorrect responses through excessive exploitation of the previous stimulus–reward association. Moreover, lOFC inactivation might enhance the capacity of the exploitation system to improve rule-based learning with new stimuli. This would predict that the new learning may be relatively impoverished and inflexible, and that, for example, subsequent reversal may be impaired.

This hypothesis raises the question of the site of interaction of the lOFC- and mOFC-dominated “systems” as the evidence of the connectivity between these OFC subregions is sparse (Price 2007; Hoover and Vertes 2011; Izquierdo 2017). It is possible that it occurs in other sites in the circuitry, for example, in the BLA (Wassum and Izquierdo 2015), or striatal–pallidal systems (Haber et al. 1995) with lOFC projecting primarily to the DLS in the rat (Heilbronner et al. 2016), whereas mOFC projects primarily to ventral striatum and dorsomedial striatum (Hoover & Vertes 2011; Heilbronner et al. 2016). It is relevant that whereas putamen inactivation in marmosets has recently been shown selectively to impair visual serial reversal learning, caudate inactivation may actually improve it (Jackson et al. 2019), which provides further evidence for a functional dichotomy in medial versus lateral circuitries in serial reversal learning.

### Effects of Inactivating mPFC (IL, PrL) on Serial Visual Reversal Learning

While the OFC subregions played critical roles selectively in the initial, perseverative phase, mPFC inactivations had rather general effects on reversal learning. IL inactivation significantly (and almost so for the PrL) reduced the number of errors to criterion irrespective of phase, supporting previous studies investigating effects of lesioning mPFC (Graybeal et al. 2011) and PrL (McAllister et al. 2015) on touchscreen reversal learning, IL-lesioning on spatial context-dependent reversal learning (Ashwell and Ito 2014) and PrL inactivation on probabilistic spatial reversal learning (however, with no effect of IL inactivation) (Dalton et al. 2016). Contrary to our results, IL-lesioned rats of Chudasama and Robbins (2003) showed an overall learning impairment (although with no effect on perseveration, as here). The different effects on learning may have arisen from the use of a rule-based serial reversal paradigm in the present study versus simple deterministic reversal learning (total of 2 reversals) in Chudasama and Robbins (2003). Thus, the findings could be understood in terms of a suppression of goal-directed behavior by the IL in favor of habitual behavior (Coutureau and Killcross 2003), the improved reversal learning following IL inactivation perhaps pointing to an underlying shift from habitual toward goal-directed behavior. This raises the obvious issue of the functional relationships among the mOFC and mPFC subregions as their manipulation produced some similarities, but also differences, in behavior. Whereas mOFC inactivation tended to mainly affect the sensitivity to immediate feedback, the mPFC manipulations had more global influences on learning performance over many trials.

### Effects of Inactivating BLA on Serial Visual Reversal Learning

Although the BLA is in general thought to play a role in reversal learning, for example, through its interaction with the OFC (Schoenbaum et al. 2002; Schoenbaum et al. 2003; Saddoris et al. 2005; Stalnaker TA, Roesch MR et al. 2007; Rudebeck et al. 2013), its specific role in reversal learning remains unresolved as studies have provided somewhat contradictory results (Stalnaker et al. 2007; Churchwell et al. 2009; Izquierdo et al. 2013). In a study most comparable to the present one, BLA lesions facilitated late reversal learning in a touchscreen visual two-choice reversal learning task with assured rewards (Izquierdo et al. 2013). One likely explanation may be linked to BLA’s role in encoding outcome-specific representations (see review by Wassum & Izquierdo 2015). The BLA is involved when an action elicits an outcome with unexpected value (Salinas et al. 1993), as also shown in reversal learning with varying outcomes (Schoenbaum et al. 2003; Churchwell et al. 2009). Oppositely, the BLA may be less involved in tasks, such as the deterministic reversal learning task, where outcome-specific representations do not confer a benefit. Thus, removing BLA’s contribution may even be an advantage enabling adaptation to a shift in contingency. Besides our results, this is supported by facilitated learning by amygdala lesions in monkeys (Rudebeck and Murray 2008) and rats (Izquierdo et al. 2013).

### Concluding Summary

This study has defined dissociable effects on visual serial reversal learning for the OFC and mPFC subregions as well as BLA that indicate separate and, in the case of lOFC and mOFC, opposite roles of these structures, depending on previous reinforcement history, that is, whether it is in the context of changing contingencies or novel discrimination. The findings are relevant to theories of PFC-dependent executive functioning and how both rodent and primate PFC mediate strategies for optimizing behavior in changing situations, which is crucial for the understanding of inflexible behavior found across different psychiatric disorders.

## Funding

Wellcome Trust Senior Investigator Grant to TWR (104631/Z/14/Z), a Lundbeck Foundation Research Fellowship (R182-2014-2810 and R210-2015-2982 to M.E.H.) and a BBSRC studentship (to L.F.).

## Notes

The experimental work was carried out under a Home Office Project License (70/7548) held by Dr A.L. Milton.
